# Identification of a Hemolysis Threshold That Increases Plasma and Serum Zinc Concentration^1^,^2^,^3^

**DOI:** 10.3945/jn.116.247171

**Published:** 2017-05-10

**Authors:** David W Killilea, Fabian Rohner, Shibani Ghosh, Gloria E Otoo, Lauren Smith, Jonathan H Siekmann, Janet C King

**Affiliations:** 4 Children’s Hospital Oakland Research Institute, Oakland, CA;; 5 GroundWork, Fläsch, Switzerland;; 6 Friedman School of Nutrition Science and Policy, Tufts University, Boston, MA; and; 7 Department of Nutrition and Food Sciences, University of Ghana, Accra, Ghana

**Keywords:** hemoglobin, hemolysis, human nutrition, mineral, plasma zinc, serum zinc

## Abstract

**Background:** Plasma or serum zinc concentration (PZC or SZC) is the primary measure of zinc status, but accurate sampling requires controlling for hemolysis to prevent leakage of zinc from erythrocytes. It is not established how much hemolysis can occur without changing PZC/SZC concentrations.

**Objective:** This study determines a guideline for the level of hemolysis that can significantly elevate PZC/SZC.

**Methods:** The effect of hemolysis on PZC/SZC was estimated by using standard hematologic variables and mineral content. The calculated hemolysis threshold was then compared with results from an in vitro study and a population survey. Hemolysis was assessed by hemoglobin and iron concentrations, direct spectrophotometry, and visual assessment of the plasma or serum. Zinc and iron concentrations were determined by inductively coupled plasma spectrometry.

**Results:** A 5% increase in PZC/SZC was calculated to result from the lysis of 1.15% of the erythrocytes in whole blood, corresponding to ∼1 g hemoglobin/L added into the plasma or serum. Similarly, the addition of simulated hemolysate to control plasma in vitro caused a 5% increase in PZC when hemoglobin concentrations reached 1.18 ± 0.10 g/L. In addition, serum samples from a population nutritional survey were scored for hemolysis and analyzed for changes in SZC; samples with hemolysis in the range of 1–2.5 g hemoglobin/L showed an estimated increase in SZC of 6% compared with nonhemolyzed samples. Each approach indicated that a 5% increase in PZC/SZC occurs at ∼1 g hemoglobin/L in plasma or serum. This concentration of hemoglobin can be readily identified directly by chemical hemoglobin assays or indirectly by direct spectrophotometry or matching to a color scale.

**Conclusions:** A threshold of 1 g hemoglobin/L is recommended for PZC/SZC measurements to avoid increases in zinc caused by hemolysis. The use of this threshold may improve zinc assessment for monitoring zinc status and nutritional interventions.

## Introduction

Zinc is an essential nutrient for normal growth, healthy pregnancy, and robust immune function. Inadequate zinc leads to stunting, developmental delays, hypogonadism, susceptibility to infections, and cognitive dysfunction (1–3). Globally, an estimated 1.2 billion individuals are at risk of zinc deficiency on the basis of national food balance sheets, with the highest prevalence in children <5 y of age (3, 4). To evaluate the zinc status of a population, nutritional surveys collect data on plasma zinc concentration (PZC)^8^ or serum zinc concentration (SZC) along with dietary information. Interestingly, these studies have indicated that the prevalence of low circulating zinc is nearly twice as high as the previous estimates of zinc deficiency from dietary or stunting data alone (5–7). Thus, the current estimates of individuals at risk of zinc deficiency could be considerably underestimated. The accurate assessment of zinc status biomarkers such as PZC and SZC is critical for understanding the true burden of zinc deficiency and evaluating the response to zinc nutrition intervention programs.

Measuring PZC and SZC requires the acquisition of blood by venipuncture or finger-stick, followed by rapid and careful processing to isolate plasma or serum for later analysis. With any study, there is a potential for variation in the processing and handling of blood samples, but especially when studies are large, multicenter, or depend on field sites with limited resources. Suboptimal blood collection and sample handling can increase the likelihood of damage to erythrocytes, resulting in some degree of hemolysis. Hemolysis has the potential to measurably increase PZC and SZC concentrations because the zinc concentration in erythrocytes is ∼10–20 times that of plasma or serum (8–10).

Hemolysis is widely recognized as a potential source of contamination or interference for a variety of hematologic variables, which has been addressed in numerous publications (11–14). It is also acknowledged that hemolysis could lead to spurious increases in PZC and SZC (6, 15–19), although there is little information as to what level of hemolysis creates a concern. Most studies that reported PZC or SZC have limited or no detailed description of how hemolysis was categorized. Therefore, the International Zinc Nutrition Consultative Group and the Biomarkers of Nutrition for Development Zinc Expert Panel have simply stated that obviously hemolyzed samples should be discarded when measuring PZC and SZC (6). More work is needed to develop evidence-based recommendations on the level of hemolysis that alters PZC and SZC. This article recommends a threshold of hemolysis for zinc analysis and compares options for assessing the degree of hemolysis. It is important to note that although plasma and serum have important physiologic differences, there does not appear to be a meaningful difference in zinc concentration between the 2 (6); consequently, the clinical reference ranges for PZC/SZC are the same (20). This article therefore treats PZC and SZC as interchangeable from the perspective of impact of hemolysis.

## Methods

### 

#### Calculations for hemolysis estimates.

The effects of hemolysis levels on PZC and SZC were estimated by using standard hematologic and mineral concentration parameters obtained from clinical reference ranges, specifically PZC and SZC (0.7–1.2 mg/L), plasma and serum hemoglobin concentration (<0.1 mg/L), erythrocyte cell concentration (3.8–5.7 × 10^12^ cells/L), erythrocyte cell volume (80–100 fL/cell), erythrocyte cell hemoglobin content (27–31 pg/cell), and erythrocyte cell zinc concentration (10–16 mg/L cell volume) (20). The degree of hemolysis needed to increase PZC and SZC values was calculated by using the equation [(*A* × *B*) ÷ (*C* × *D*) ÷ *E*] × 100 = *F*, where *A* is the average PZC and SZC of the study or population, *B* is the measure of precision beyond which zinc values are considered increased over the mean PZC and SZC, *C* is the average erythrocyte zinc concentration of the study or population, *D* is the average erythrocyte volume (also known as mean corpuscular volume) of the study or population, and *E* is the average erythrocyte concentration (also known as RBC count) of the study or population. The product (*F*) of this equation is the percentage of erythrocyte lysis required to yield the desired increase in PZC and SZC. The concentration of hemoglobin that corresponds to the determined degree of hemolysis was calculated according to the equation *G* ÷ [(*A* × *B*) ÷ (*C* × *D*)] = *H*, where *A*–*D* represent the same values as above and *G* is the erythrocyte hemoglobin concentration (also known as mean corpuscular hemoglobin content). The product (*H*) of this equation is the concentration of hemoglobin that indicates a significant increase in PZC and SZC.

#### In vitro hemolysis study.

Blood samples from 14 healthy participants (**Supplemental Table 1**) were purchased from a commercial laboratory (AllCells LLC). The laboratory prescreened donors for lack of infectious agents (e.g., HIV, hepatitis B virus, and hepatitis C virus), chronic illness, and medication or supplement use within the previous 2 wk. Venous whole blood was collected in trace metal–certified Vacutainers containing lithium heparin as anticoagulant (BD), inverted several times, and stored at room temperature until processing within 2 h. Whole blood was centrifuged at 600 ×* g* for 15 min at room temperature followed by immediate removal of the plasma. The plasma was transferred to polypropylene microtubes (tested free of metal contamination) and further centrifuged at 3000 × *g* for 5 min at room temperature to remove any remaining cells and debris. The clarified plasma was then transferred to new polypropylene microtubes and stored at −80°C. For concentrated hemolysate, erythrocyte-rich plasma was allowed to gradually hemolyze over 4 mo at 4°C to reduce the formation of precipitate or insoluble material that could affect mineral concentration. To generate graded hemolysis, defined amounts of concentrated hemolysate were added to nonhemolyzed plasma and mixed to uniformity at 6 different ratios of 0%, 0.1%, 0.25%, 1%, 2.5%, and 10% concentrated hemolysate volume into control nonhemolyzed plasma volume. Ten experiments were completed from the participant samples for a total of 60 independent points. Graded hemolysis samples were then analyzed for hemoglobin and mineral concentrations. The commercial laboratory also provided basic anthropometric and hematologic donor information, but no protected health information, so institutional review board (IRB) registration was not required.

#### Population nutritional survey.

Briefly, whole blood was collected from 909 infants at 6–18 mo of age living in Ghana for the 2012–2013 Trial for Reducing Undernutrition through Modified Feeding study (21). Blood was collected in trace metal–certified Vacutainers without anticoagulant (BD) at multiple locations, transported back to a central storage facility, separated into serum samples, and stored at −80°C. In 2016, serum samples were shipped in batch to the Children’s Hospital Oakland Research Institute for the determination of SZC. At the Children’s Hospital Oakland Research Institute, serum samples were thawed, mixed on a vortex for 5 s, and prepared for mineral analysis as described below. Before proceeding with elemental analysis, each sample was compared visually to a hemolysis color scale by the same trained technicians and assigned to 1 of 6 hemolysis levels from 0 to 10 g hemoglobin/L. Additional description and results from this population-based study will be described in a future study (S Ghosh, unpublished data, 2017). The study was monitored by the host institute IRB; no protected health information was communicated for elemental and hemolysis analysis, so additional IRB registration was not required.

#### Mineral analysis.

Iron and zinc concentrations were determined by inductively coupled plasma optimal emission spectrometry (ICP-OES) as previously described (22, 23). For PZC and SZC, 50- to 100-μL samples were removed from thawed stocks and processed for mineral analysis. The ICP-OES was calibrated by using National Institute of Standards and Technology traceable elemental standards (Sigma-Aldrich) and validated by using Seronorm Trace Element Levels 1 and 2 reference material (Sero). The detection range for both iron and zinc was between 0.005 and 5.000 mg/L and the CV for interassay precision was <5%. Cesium (50 mg/L) was used for ionization suppression, and yttrium (5 mg/L) was used as an internal standard for all samples. All associated reagents and plasticware were certified as trace metal free or tested for trace metal contamination. Iron and zinc concentrations were normalized per plasma or serum volume.

#### Determination of hemoglobin concentration.

Hemolysis is commonly quantified by the corresponding amount of hemoglobin released into the plasma or serum. The concentrations of hemoglobin were determined by using the following: *1*) direct chemical detection of hemoglobin with the use of Drabkin’s assay according to standard protocols (24); *2*) indirect chemical detection of hemoglobin with the use of iron concentration calculated from standard molar ratios (4 mol Fe/1 mol hemoglobin); *3*) spectroscopic estimation of hemoglobin by directly measuring absorbance of plasma or serum at 540 nm, a major absorbance peak for hemoglobin; or *4*) visual inspection of plasma or serum with the use of a hemolysis color scale generated by adding defined amounts of hemoglobin from a human hemoglobin standard (Point Scientific) to fresh plasma or serum containing no apparent hemolysis (hemoglobin <0.2 g/L and no orange-red tint). For spectroscopic estimation, 100 μL plasma or serum was pipetted into 96-well flat-bottom polystyrene microplates and measured on a conventional spectrophotometer with path-length correction (Synergy H1; Biotek Instruments); background absorbance of empty wells was ∼0.05 absorbance units. For visual inspection, 3 independent color scales were generated in optically clear plastic cuvettes and imaged with a digital camera. Color was determined by using Adobe Photoshop Elements color picker tool (version 13.1) with all files converted to Adobe RGB color profile (with a gamut better matched to human visual perception) on a computer monitor with a calibrated color spectrum. Color values were determined by using the color picker tool to aggregate ∼50% of the color image with the use of an 8-bit red, green, blue (RGB) color model. RGB values can be converted to other color models for printing by using printers with appropriate color-monitoring tools.

#### Statistical analysis.

Graphing, regression, and statistical analysis were conducted by using Prism software, version 6 (GraphPad Software, Inc.). For comparisons of *1*) in vitro plasma mineral and hemoglobin concentrations, *2*) in vitro plasma zinc and plasma iron concentrations, and *3*) population serum zinc and zinc and serum iron concentrations, linear regression was used. For comparison of population serum mineral concentrations with hemolysis range, 1 data point was removed from both the iron and zinc values because they were >3 SDs from the hemolysis range group mean, which left 908 values for each mineral. The mean serum iron and zinc concentrations for each hemolysis range were compared by using a 1-factor ANOVA with Dunnett’s multiple-comparison ad hoc test for differences compared with the control (nonhemolyzed) group. Serum mineral concentration data did not pass the test for normal distribution (D’Agostino and Pearson omnibus test, α = 0.05), but sample sizes were large enough for the ANOVA model to be robust. For all of the tests, significance was accepted at *P* < 0.05.

## Results

### 

#### Effects of hemolysis on PZC or SZC by calculation.

The degree of hemolysis required to increase PZC and SZC was estimated by using values within the standard hematologic and mineral concentration reference ranges. To model these calculations for a population in whom nutrient deficiencies are common, lower values for each parameter were used. In addition, the analytical techniques that measure PZC and SZC, including atomic absorption spectrometry (AAS) or ICP-OES, typically have a precision of ∼5% CV (25). Therefore, the target amount of hemolysis required to detect an increase in PZC and SZC was selected as 5%. In practice, the population values or desired precision may vary, so the parameters can be easily adjusted as needed.

The calculations to determine a threshold level of hemolysis use the equations described in Methods on the basis of the following descriptions of each step:

1. Determine the amount of zinc needed to increase PZC and SZC by 5%. For an average PZC and SZC of 0.7 mg/L, an additional 0.035 mg Zn/L would be released into the plasma or serum.
2. Determine the fraction of erythrocyte zinc that would increase PZC and SZC by 0.035 mg/L. For an average erythrocyte zinc concentration of 10 mg/L and erythrocyte volume of 80 fL/cells, the erythrocyte zinc content would be 0.8 fg/cell. To release 0.035 mg Zn into the plasma or serum, the concentration of lysed erythrocytes would be 4.375 × 10^10^ cells/L.
3. Determine the level of hemolysis that equates to 4.375 × 10^10^ erythrocytes/L. For an average erythrocyte concentration of 3.8 × 10^12^ cells/L, 4.375 × 10^10^ cells/L represents 1.15% of the erythrocyte concentration.
4. Determine the concentration of hemoglobin that equates to 1.15% of erythrocytes. For an average erythrocyte hemoglobin concentration of 27 pg/cell and an erythrocyte concentration of 4.375 × 10^10^ cells/L, 1.12 g hemoglobin/L would be released into the plasma or serum. Because normal plasma or serum concentrations of hemoglobin are negligible, then 1.12 g hemoglobin/L represents the actual hemoglobin that would be measured by lysis of 1.15% of erythrocytes.

#### Effects of hemolysis on PZC in vitro.

**FIGURE 1:**
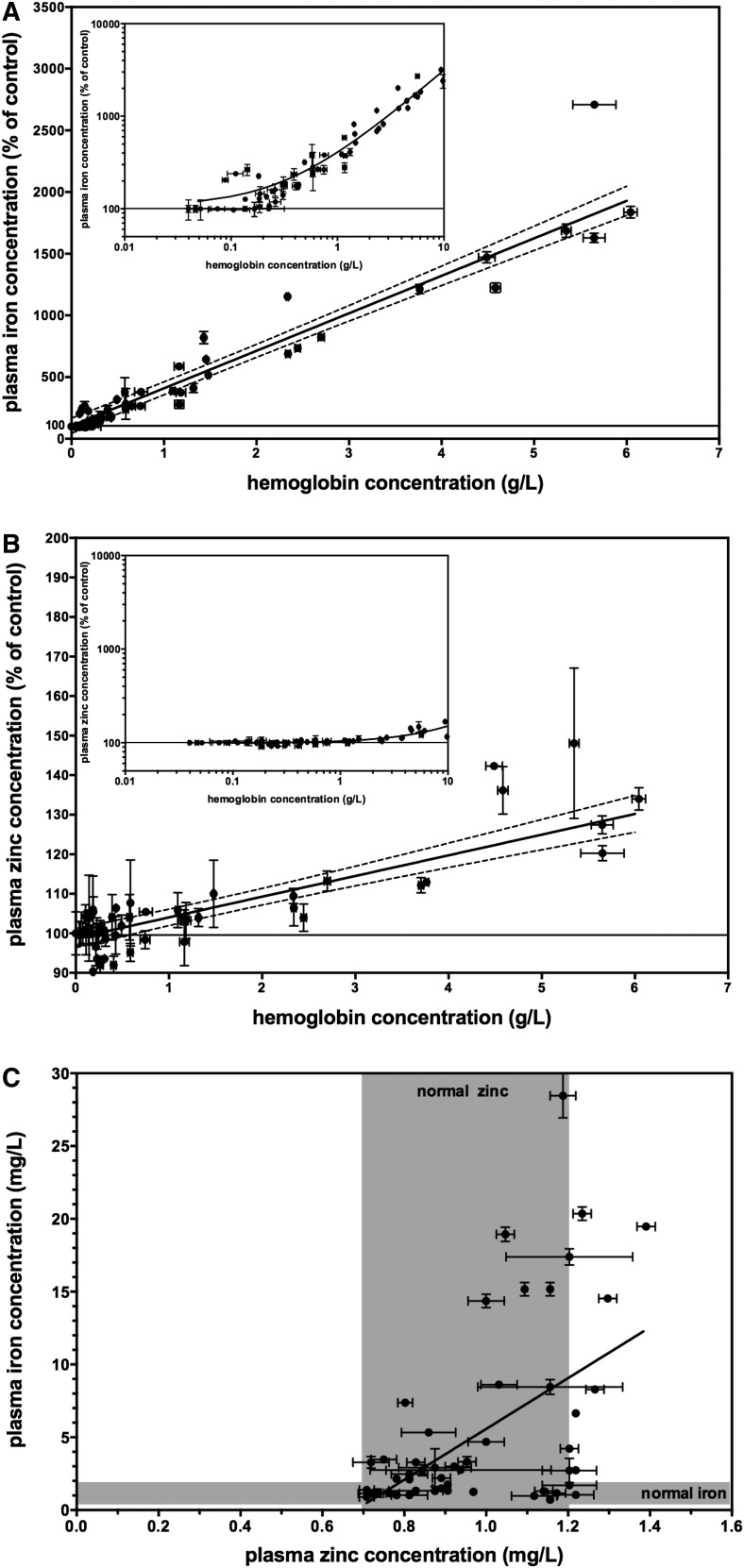
Effects of hemolysis on plasma iron and zinc concentrations in vitro. The trace mineral concentration increased as a function of hemolysis as indicated by the hemoglobin concentration. (A) Plasma iron concentrations increased with the addition of hemolysate to nonhemolyzed plasma. Values are means ± SDs, *n* = 60. Data were fit to a linear function (solid line, *R*^2^ = 0.918) with a 95% confidence band (dashed line) also shown. The inset shows data on a log_10_ scale. (B) Plasma zinc concentrations increased with the addition of hemolysate to nonhemolyzed plasma at ∼1 g hemoglobin/L. Values are means ± SDs, *n* = 60. Data were fit to a linear function (solid line, *R*^2^ = 0.698) with a 95% confidence band (dashed line) also shown. The inset shows data on a log_10_ scale. (C) Iron and zinc concentrations were plotted for each plasma sample. Gray shading indicates the clinical reference range for plasma iron and zinc concentration as indicated (20). Values are means ± SDs, *n* = 60. Data were fit to a linear function (solid line, *R*^2^ = 0.274).

To empirically determine the level of hemolysis that would increase PZC by 5%, concentrated hemolysate was added to nonhemolyzed plasma followed by hemoglobin and mineral analysis. Ratios of hemolyzed to nonhemoylzed plasma were chosen both within and exceeding the clinical reference range for iron (0.5–1.75 mg/L) and zinc (0.7–1.2 mg/L) concentrations in plasma or serum (20). Iron concentration increased with the addition of hemolysate, reaching nearly 3000% of the control value as hemoglobin concentrations approached 10 g/L (**Figure 1A**). The data fit a linear function *y* = 303.9 (*x*) + 105.4 with an *R*^2^ of 0.918. In contrast, the zinc concentration did not consistently change at lower levels of added hemolysate, but increased modestly only after the hemolysate level reached 1 g hemoglobin/L in the plasma (Figure 1B). The data fit a linear function *y* = 5.233 (*x*) + 98.80 with an *R*^2^ of 0.698. The best-fit line for the zinc response crossed the level of 5% over baseline at a hemoglobin concentration of 1.18 ± 0.10 g/L, similar to findings from the calculated values. However, the correlation between iron and zinc values within the same sample was weak; the linear function fit the equation *y* = 17.44 (*x*) − 11.86 with an *R*^2^ of 0.274 (Figure 1C).

#### Effects of hemolysis on SZC in a population study.

**FIGURE 2:**
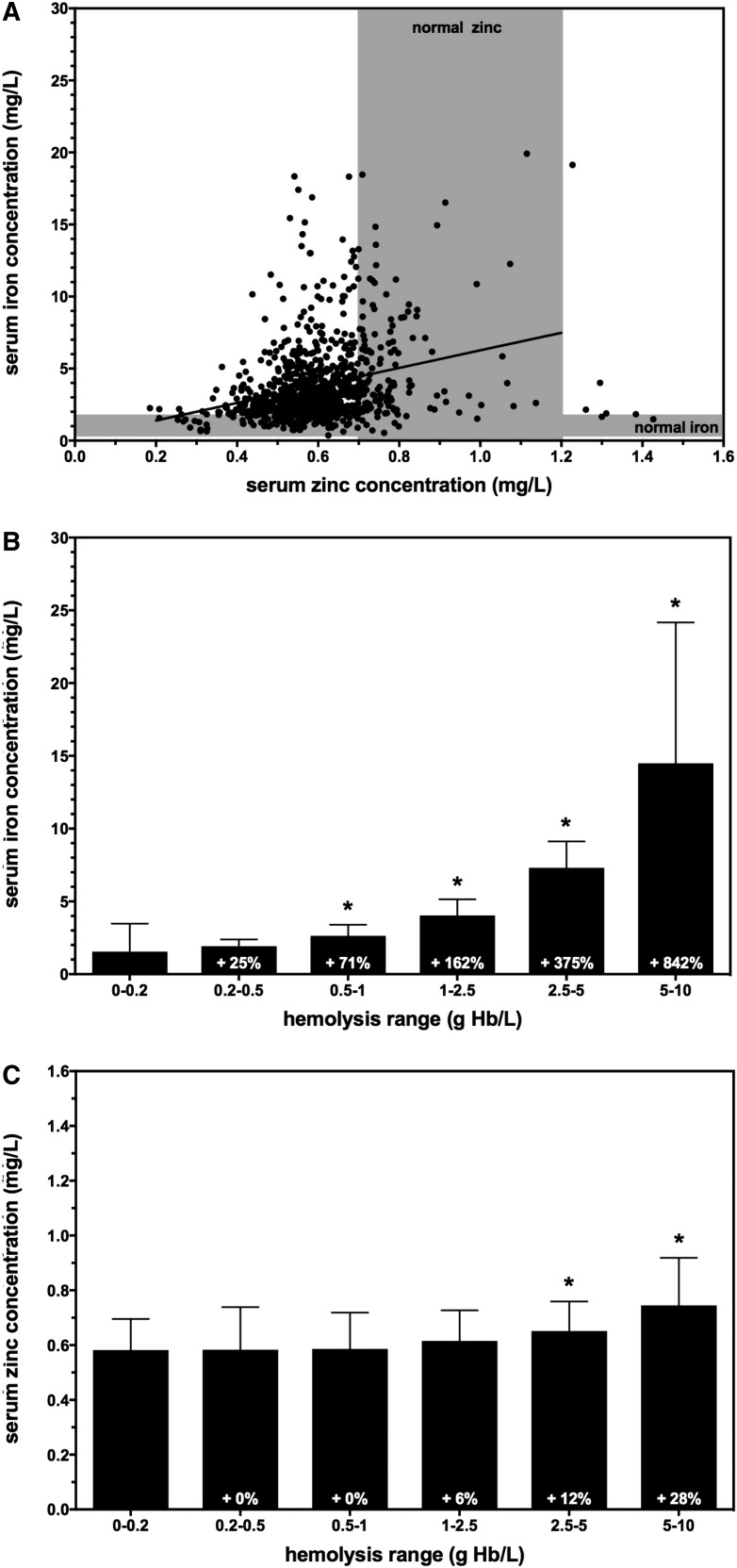
Effects of hemolysis on serum iron and zinc concentrations in a population nutritional survey. (A) Iron and zinc concentrations were plotted for each serum sample. Gray shading indicates the clinical reference range for serum iron and zinc concentrations as indicated (20). Data were fit to a linear function (solid line, *R*^2^ = 0.104). (B) Serum iron concentrations increased as a function of hemolysis range grouping on the basis of hemoglobin concentration. Values are means ± SDs, *n* = 46–289. (C) Serum zinc concentrations increased as a function of hemolysis range grouping on the basis of hemoglobin concentration. Values are means ± SDs, *n* = 46–288. *P < 0.05. Hb, hemoglobin.

To test the hemolysis threshold of 1 g hemoglobin/L, the correlation between apparent hemolysis level and SZC was examined in samples obtained in a large population study conducted in Ghana. SZCs ranged from 0.21 to 1.44 mg/L, with a mean ± SD of 0.61 ± 0.13 mg/L (**Figure 2A**). SZCs were low compared with the reference ranges in the United States, but this was expected because 15–25% of the population in Ghana is estimated to be at risk of zinc inadequacy (4). Mineral analysis was conducted by ICP-OES, so serum iron concentrations were also determined. Serum iron concentrations ranged from 0.36 to 72 mg/L, with a mean ± SD of 3.92 ± 3.76 mg/L (Figure 2A). Many (∼40%) samples exceeded the clinical reference range for serum iron concentration and had a visible tint ranging from light orange to dark red, suggesting elevated hemolysis levels in those samples. When the mineral values were plotted together, the data fit a linear function *y* = 6.087 (*x*) + 0.1793 with an *R*^2^ of 0.104, indicating a weak correlation between serum iron and zinc values.

The samples were then divided into 6 groups on the basis of apparent hemolysis level: 0–0.2, 0.2–0.5, 0.5–1, 1–2.5, 2.5–5, and 5–10 g hemoglobin/L. Compared with the 0–0.2-g/L (baseline) group, mean serum iron concentrations in the other groups increased markedly from 25% to 842%, which is consistent with increasing hemolysis (Figure 2B). Zinc concentrations were then assessed to determine which hemolysis group would reach the target of 5% above baseline SZC. The 0.2–0.5- and 0.5–1-g/L hemolysis groups had the same mean SZC as the baseline group, whereas the 2.5–5- and 5–10-g/L hemolysis groups had mean SZCs 12% and 28% higher than baseline, respectively (Figure 2C). The 1–2.5-g/L hemolysis group showed the closest match to the 5% target, with a mean SZC of 6% over baseline, although this did not reach significance. However, the measured 6% increase in mean SZC for the 1–2.5-g/L group is consistent with the trend of elevated SZC in the groups with greater hemolysis and was consistent with a 5% increase in zinc concentrations from 1 g hemoglobin/L hemolysates as determined above. It is important to note that this analysis is based on the assumption that mean SZC would be the same between all groups without the addition of hemolysis, which was not possible to test independently.

#### Evaluation of hemoglobin concentrations to assess hemolysis in plasma or serum.

**FIGURE 3:**
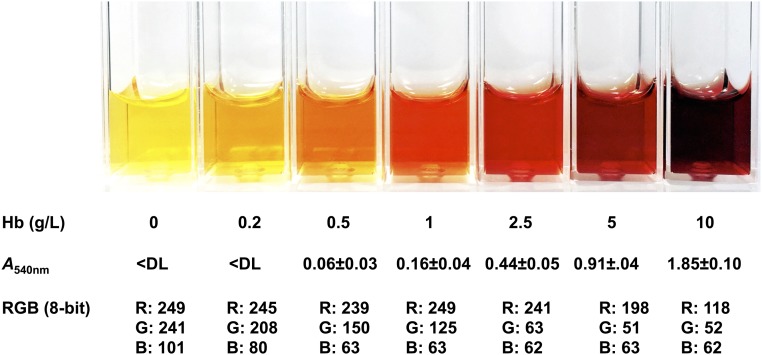
Methods to determine hemolysis levels in plasma or serum. A representation of plasma or serum within increasing levels of hemolysis is shown. Below the images are the corresponding Hb concentrations (means ± SDs; *n* = 3) and approximate RGB color model values of the corresponding plasma or serum. The iron values listed for the lowest category of hemolysis reflect the clinical reference range in nonhemolyzed plasma or serum (0.5–1.8 mg/L) (20). *A*_540nm_, absorbance at 540 nm; Hb, hemoglobin; RGB, red, green, blue; <DL, below the detection limit of 0.05 absorbance.

Several approaches were used to determine hemoglobin concentrations in plasma or serum. Chemical detection of hemoglobin has a typical minimum detection limit of ∼0.01 g/L (24), but these assays are often not convenient for large, multicenter, and/or field studies. One alternative approach is the use of a simple color scale to estimate the degree of hemolysis (**Figure 3**). The apparent color of 1 g hemoglobin/L in plasma or serum is distinctly orange-red, which is similar to an RGB color model value of 249, 125, 63. If greater precision is needed, direct measurement of the absorption of plasma or serum samples was found to be sufficient to detect 1 g hemoglobin/L, without chemical modification of the constituent hemoglobin (**Supplemental Figure 1**). Hemoglobin has several major absorption peaks with varying sensitivity. However, plasma or serum had a significant absorbance at the smaller wavelength peaks, so the absorbance peak at 540 nm is recommended. The absorbance value for 1 g hemoglobin/L at 540 nm was 0.16 ± 0.04.

## Discussion

Hemolysis can affect the constituent analysis of serum or plasma. For zinc measurement, this can be a concern because the zinc concentration in erythrocytes is 10–20 times that of plasma or serum (8, 10, 20). Many studies for measuring PZC and SZC simply warn the investigator to avoid hemolysis, without providing specific quantitative metrics. Underestimating the impact of hemolysis can result in the inclusion of samples containing plasma or serum contaminated with inflated amounts of zinc, which adds noise to the analysis of zinc status in a population. Overestimating the impact of hemolysis can result in the rejection of samples that are perfectly acceptable for zinc measurement, and thus losing study power and introducing sampling bias. Having a clear threshold of hemolysis for measuring PZC and SZC is important for evaluating zinc status and response to zinc nutrition interventions.

In this study, we approached this problem in 3 different ways: *1*) theoretical calculation by using standard hematologic values and mineral concentrations, *2*) controlled addition of hemolysate in vitro, and *3*) correlation of zinc concentration to hemolysis levels in samples from a large population study. All 3 approaches indicated a similar value of ∼1 g hemoglobin/L as the level of hemolysis that increased PZC and SZC by ∼5%. This target level of increased zinc was chosen because the analytical techniques that measure PZC and SZC, such as AAS or ICP-OES, typically yield a CV of 5% (25). Therefore, a 5% increase in PZC or SZC would be a minimum value to reliably detect changes in PZC and SZC. However, it would be straightforward to adjust these calculations if a different percentage increase in PZC and SZC was preferred. For example, the WHO estimates that >1 billion women and children in developing countries suffer from iron deficiency and anemia (26); therefore, the concentrations of erythrocyte hemoglobin and other hematologic values in these areas are likely to be even lower than the example values used in this analysis. The degree of hemolysis can be determined by using the same calculations as above, replacing the specific hematologic and mineral concentration parameters for the desired study population.

Several reports measuring PZC and SZC mentioned the potential confounding effects of hemolysis, yet do not provide details as to how samples with observable hemolysis were evaluated. Other studies have stated that hemolyzed samples were recorded and/or discarded, but do not indicate the level of hemolysis used for threshold. Only a few studies, to our knowledge, directly investigated the effect of hemolysis on PZC/SZC, but the methodologies varied and often used descriptive analysis of hemolysis (e.g., low, moderate, and extensive) without specific quantitative values (27, 28). Lofberg and Levrl (15) reported no significant change in SZC in a small study of “hemolyzed” compared with “unhemolyzed” samples, but estimated that a “1–2% hemolysis” could result in increased zinc. Strand et al. (27) measured the changes in zinc from hemolyzed samples but with the use of qualitative categorization (mild, moderate, or extensive) measured by trained technicians. However, the understanding of these categories may differ between laboratories.

Measuring hemoglobin concentrations in plasma or serum is arguably the most convenient way to determine the level of hemolysis in a blood sample, because the normal concentration of hemoglobin in plasma or serum is negligible compared with the amount of hemoglobin released during even mild hemolysis. There are several ways to measure hemoglobin. The classic chemical method involves the transformation to methemoglobin followed by reaction with alkaline potassium cyanide (24). Improved versions of the chemical method for detecting hemoglobin are now available that avoid the use of toxic cyanide compounds and extend the sensitivity of the assay (29). In addition, point-of-care devices that directly measure hemoglobin in small blood samples are available and now commonly used in field studies (30). Strengths of the chemical and device-based measurements include a straightforward protocol and high sensitivity. Weaknesses of this approach include the additional time and procedural steps, increased costs, and use of toxic chemicals for the chemical assays; these complications are particularly inconvenient for large, multicenter, and/or field studies. In addition, the high sensitivity of these assays is not necessary to measure the relatively high threshold level of 1 g hemoglobin/L.

Therefore, alternative ways to assess hemoglobin concentration were investigated that might be more amenable for large and/or field studies of PZC and SZC. Because hemoglobin has significant absorbance within the visual spectrum, hemoglobin in plasma or serum can be detected by direct spectrophotometry without any chemical modifications. This method is less sensitive than the chemical methods, but is easily able to detect hemoglobin at 1 g/L. Strengths of this approach include minimal additional costs, a simple protocol, and a nondestructive method that allows the plasma or serum to be used for other measurements. Weaknesses of this approach include additional time and procedural steps and inability to resolve color variations in plasma unrelated to hemolysis, such as infection or high concentrations of bilirubin, carotenoids, or ceruloplasmin (31).

Another proposed way to assess hemoglobin concentration is to take advantage of the constitutive iron within the hemoglobin protein. If technologies such as ICP-OES are used to measure PZC and SZC, then iron concentration can be measured in the same analytical run. The additional iron added into plasma or serum by 1 g hemoglobin/L is substantially greater than endogenous concentrations, and thus might be useful to identify samples that exceed the hemolysis threshold. Strengths of this approach include that no additional time, procedural steps, or costs are needed beyond what is needed to determine PZC and SZC. Weaknesses of this approach include the inability to distinguish between hemolysis and elevated iron resulting from iron supplementation or contamination of samples. In addition, ICP-OES and similar technologies are not always available to researchers; other common technologies such as AAS can also measure iron but require separate analyses with additional sample material. Furthermore, we found that in the hemolysis analysis in both the in vitro plasma (Figure 1C) and the population serum (Figure 2A) samples, there was only a weak correlation between iron and zinc concentrations in the plasma or serum. Therefore, we do not recommend iron concentrations alone as an approach to screen plasma or serum samples for PZC and SZC analysis.

The final way to estimate hemoglobin concentration is to visually compare the plasma or serum samples to a hemolysis color scale, based on the fact that hemoglobin is strongly colored. This approach has been reported in other publications (11, 14). Strengths of this approach include that it has no additional procedural steps or costs. This technique is easy to use, even for large-population studies, and simple to teach the technical staff. Weaknesses of this approach include the need for some additional time and the lack of quantitative hemoglobin measurement. However, we found that, once trained, technical staff are able to easily and rapidly identify plasma or serum samples that exceed the color threshold. To facilitate this approach, we created a simple card containing a hemolysis index with identification of our recommended threshold of hemolysis. This card is available on the International Zinc Nutrition Consultative Group website (www.izincg.org) and can be digitally displayed (e.g., on a smartphone or tablet) or printed, assuming that color-management tools are used to correct for variance in printer output.

Once the relation between hemolysis and PZC and/or SZC is clearly defined, there might be interest in a correction factor that could remove the zinc concentrations originating from erythrocyte leakage from PZC and SZC calculations. For example, this was a common approach for the measurement of potassium concentrations in plasma or serum (32). However, we do not recommend this approach for PZC and SZC due to the substantial variance found in erythrocyte zinc concentrations, hemoglobin concentrations, and other factors. Recently, this approach has become increasingly criticized even for the determination of potassium, which tends to be less variable than PZC and SZC (33).

This study recommends a discrete threshold of hemolysis and hemoglobin concentration beyond which the plasma or serum concentrations of zinc could be compromised due to leaching of erythrocyte zinc stores. This conclusion was reached through a combination of theoretical calculations, simulated hemolysis testing in vitro, and analysis of samples from a large field study. There are many other minerals, vitamins, and enzyme markers in which plasma or serum concentrations could be compromised by hemolysis. By using similar approaches to this study, appropriate thresholds could be reached for all of these nutrients or biomarkers.

## Footnotes

8 Abbreviations used: AAS, atomic absorption spectrometry; ICP-OES, inductively coupled plasma optical emission spectrometry; IRB, institutional review board; PZC, plasma zinc concentration; RGB, red, green, blue; SZC, serum zinc concentration.
