# Corrigendum to: Identification of a Hemolysis Threshold That Increases Plasma and Serum Zinc Concentration. J Nutr 147;6: 1218–25

**DOI:** 10.1093/jn/nxab121

**Published:** 2021-06-01

Address correspondence to DWK (e-mail: david.killilea@ucsf.edu).

Corrigendum to: Identification of a Hemolysis Threshold That Increases Plasma and Serum Zinc Concentration. J Nutr 147;6: 1218–25.

In the original article, errors were noted and have been listed in this corrigendum. These details have been corrected only in this corrigendum to preserve the published version of record.

In the Methods section, for the “Calculations for hemolysis estimates” subsection, the formula *G* ÷ [(*A* x *B*)÷(*C* x *D*)] = *H* has an incorrect operator. The formula should be written as *G* x [(*A* x *B*)÷(*C* x *D*)] = *H*, so the first obelus should become a times sign. Therefore, the following text should read: 

“The concentration of hemoglobin that corresponds to the determined degree of hemolysis was calculated according to the equation *G* x [(*A* x *B*)÷(*C* x *D*)] = *H*, where *A*–*D* represent the same values as above and *G* is the erythrocyte hemoglobin concentration (also known as mean corpuscular hemoglobin content). The product (*H*) of this equation is the concentration of hemoglobin that indicates a significant increase in PZC and SZC.”

In the Acknowledgments, the following text should read: 

“We thank Tatiana Cheong, Darryl Chow, Caralyn Gonzales, Wesley Kwong, and Kathleen Schultz for technical assistance and Kenneth Brown, Christine McDonald, and Bradley A. Woodruff for helpful comments on this study.”

